# Social participation and independence in activities of daily living: a cross sectional study

**DOI:** 10.1186/1471-2318-9-26

**Published:** 2009-07-07

**Authors:** Encarnación Rubio, Angelina Lázaro, Antonio Sánchez-Sánchez

**Affiliations:** 1 Department of Microbiology, Preventive Medicine and Public Health, Faculty of Medicine, Domingo Miral, s/n. 50009, University of Zaragoza, Zaragoza, Spain; 2 Department of Applied Economics, Faculty of Law, University of Zaragoza, Spain; 3 Department of Applied Economics, Faculty of Economics, University of Zaragoza, Zaragoza, Spain

## Abstract

**Background:**

It is today widely accepted that participation in social activities contributes towards successful ageing whilst, at the same time, maintaining independence in the activities of daily living (ADLs) is the *sine qua non *for achieving that end. This study looks at people aged 65 and over living in an urban area in Spain who retain the ability to attend Social Centres providing recreational facilities. The aim of this paper is to quantify independence and identify the risk factors involved in its deterioration.

**Methods:**

The sample size was calculated using the equation for proportions in finite populations based on a random proportional sample type, absolute error (e) = 0.05, α = 0.05, β = 0.1, p = q = 0.5. Two-stage sampling was used. In the first place, the population was stratified by residence and a Social Centre was randomly chosen for each district. In the second stage, individuals were selected in a simple random sample without replacement in proportion to the number of members at each social centre.

A multivariate logistical regression analysis takes functional ADL capacity as the dependent variable. The choice of predictive variables was made using a bivariate correlation matrix. Among the estimators obtained, Nagelkerke's R^2 ^coefficient, and the Odds ratio (CI 95%) were considered. Sensitivity and 1-specificity were adopted to present the results in graphic form.

**Results:**

Out of this sample, 63.7% were fully capable of carrying out ADLs, while the main factors contributing to deterioration, identified on the basis of a logistic regression model, are in order of importance, poor physical health, poor mental health, age (above 75 years) and gender (female). The model employed has a predictive value of 88% and 92% (depending on the age range considered) with regard to the independence in ADLs.

**Conclusion:**

A review of the few Spanish works using similar methodology shows that the percentage of non-institutionalised persons who are independent enough to carry out ADLs is considerably lower than that found in this study of socially-active persons. Participation in recreational activities as part of a community may delay the onset of the dependence associated with ageing.

## Background

Life expectancy has increased remarkably in almost all European countries in recent decades. However, it is not clear whether the years gained are years in good health or years lived with disability and in need of help [1]. In this light, the quality of the extra years of life is more important than overall life expectancy [2]. Indeed, 'adding life to the years' has become a key concept of 'successful ageing' [3], itself a prominent theme in contemporary applied gerontology [4].

The concept of successful ageing, introduced by Rowe and Kahn, implies that those ageing successfully would present the combination of low probability of disease and of deficiencies related to diseases, maintenance or strengthening of physical and cognitive functions and full engagement in life, including productive activities and interpersonal relationships [5-7]. These interdependent factors differentiate "normal ageing" from "successful ageing".

Another psychological perspective interprets successful ageing as a general process of adaptation, described as selective optimisation with compensation (SOC). This model presupposes that the three elements -selection of goals because not all opportunities can be pursued, optimisation or enrichment of reserves and resources, and compensation or utilisation of alternative means to reach the same goal- constitute the basic component processes for changes regarding ageing and adaptive capacity, and that the three always interact [8].

Meanwhile, the World Health Organisation has introduced the close concept of "active ageing" [9], which it defines as "the process of optimising opportunities for health, participation, and security in order to enhance the quality of life as people age". This definition refers not only to staying physically active but also "to continuing participation in social, economic, cultural, spiritual and civil affairs" [10]

While the notion of successful ageing is an attractive one, it has proven difficult to operationalise [11]. Some authors define subjects that are ageing successfully as those who score highest on a combined scale of physical function and exercise [12], such as those with minimal interruption of usual functioning, although minimal signs and symptoms of a chronic condition may be present [11]; with good physical and mental function (with a reduced number of chronic conditions), or good mobility, good cognitive function and absence of depression, and capacity to live an independent life [13]; or having a superior quality of life under a subjective perspective [14].

There appears to be no agreement on how to measure successful ageing. Given that as people age their quality of life is largely determined by their ability to maintain autonomy and independence [15,16], the absence of limitation in performing ADLs plays an important role [4,14,17]. In this context, the capacity to perform the activities of daily living appears to be a *sine qua non *for successful ageing. Thus, being fully functional in the activities of daily living, or having "no disability in performing ADLs", has been adopted as the objective indicator of successful ageing.

There is growing recognition today that social engagement is relevant for successful ageing [18]. Participation in social activities promotes physical health [19], mental health [20], intellectual functioning [21] and survival [22-24]. On the other hand, social activity is associated with better physical functioning, a lower risk of future dependence and functional recovery in order to perform ADLs [25-27].

Social engagement can be interpreted as community involvement, for example, in terms of membership of neighbourhood associations, religious groups or non-governmental organisations. Community involvement is occasionally referred to as formal social relations, compared to informal social relations, a term covering ties to family and friends. Additionally, social networks refer to structural aspects of social relations, comprising both the fabric of individuals with whom one maintains interpersonal relations and the characteristics of the ties (in terms of the number of ties, proximity of relationship, frequency of contact, reciprocity or duration) [28]. This definition permits an approach to be made to social relations on the whole, although there is no consensus on the ideal instrument to measure these relations. An excellent review of the beneficial effects of social relations on several health issues can be seen in the work of Zunzunegui et al. [27].

This paper looks at elderly people living at home in an urban area in Spain. One feature of the sample population is that these people habitually use local recreational facilities. They therefore do participate in social life in the sense of "time spent in social interaction" [24], as the social centres concerned organise activities such as music and theatre groups, card games, and introductory computer courses, among others. To the best of our knowledge, the target population selected for the study is novel in the existing literature. We would argue this because it is made-up of individuals with a high level of independence, given that all participants are still living at home and they use the social resources already present in the community by frequenting social centres. These centres were set up with the treble purpose of encouraging preventive activity that would allow members' physical, psychological and social conditions to be maintained; to create useful support services and to provide channels for community involvement.

Based on this sample population, the aim of this study is to quantify the absence of dependence in performing the basic and instrumental activities of daily life and to identify the factors contributing to functional dependence for older people.

## Methods

### Population and sample

This study, which forms part of a wider research project entitled 'Old Age and Dependency in Aragon (Spain)', looks at a target population of people aged 65 or over who live at home and regularly attend social centres. This population comprises 53,632 people in Zaragoza, a city in northeastern Spain with close to 700,000 inhabitants.

The sample size was calculated using the equation for proportions in finite populations, based on a random proportional sample type, absolute error of e = 0.05, α = 0.05, β = 0.1, p = q = 0.5, requiring a degree of precision of over 90%. Having excluded questionnaires containing seven or more errors (i.e. questionnaires in which no response was given to a number of essential questions or containing inconsistencies in the responses given to similar questions) in a preliminary stage, the sample size was 380. Given that the smallest volume of responses to each individual question in the questionnaire was 348, the minimum level of precision is 91.3%.

Two-stage sampling was used. In a first step, the population was stratified by area of residence and a social centre was chosen at random for districts with more than one social centre. Each social centre was assigned a sample size in proportion to the number of members. The second step consisted of stratified sampling for each social centre with proportionate allocation by sex and age, and morning or afternoon/evening visits. Sample units were chosen by simple random sampling. If a selected individual expressed unwillingness to form part of the study, he/she was replaced by another of similar characteristics.

### Data gathering

The questionnaire used was the validated and adapted Spanish language version [29,30] of the OARS-MAFQ (Older Americans Resources and Services Program-Multidimensional Functional Assessment Questionnaire) or OARS [31,32]. The OARS questionnaire was chosen because it provides functional assessment of the non-institutionalised elderly persons in five dimensions: social resources, economic resources, mental health, physical health and the capacity to perform ADLs. It therefore allowed a large amount of information to be gathered, although not all was used in this study. The survey was carried out through face-to-face interviews. For the purposes of this study, the variables selected from the exhaustive OARS consisted, in addition to descriptive variables for the population, on those suitable to assess functional capacity in the area of ADLs, personal finances, and mental and physical health. With respect to perceived health, this variable has been classified as good and normal-poor.

### Socio-demographic variables

The age of the interviewees was recoded in two categories and in two different ways. Thus, the sample was classified into persons aged under 75 years or 75 and older, and into persons aged under 80 years or 80 and older. In the first place, this choice reflects the fact that the percentage of individuals in the oldest age groups is small, given the characteristics of the population studied. Secondly, a more detailed examination of the survey reveals that it is precisely in this 5-year period where the point of inflexion is located with respect to the use of social and health services and, therefore, we may infer that this is also the case in respect to dependency. The level of education was classified as incomplete primary schooling or primary or higher schooling. The three groups established for marital status were, unmarried-separated-divorced, married (including those living in a stable partnership) and widowed. The latter were not included in the first group because we observed that their behaviour was different and that they required more assistance.

### Measurement of personal finances

Personal finances were measured by scoring 16 questions. These referred directly to the pension actually received and whether the participant had any other sources of income. Other indirect questions referred to the individuals' occupation, the characteristics of their dwelling and whether they could afford occasional treats or not.

### Measurement of mental and physical health

Mental health was assessed through six questions, one of them being the Short Psychiatry Evaluation Schedule [33,34]. The participants' general concerns were also considered, along with their appraisal of life as routine, boring or interesting.

Nineteen questions were asked to measure physical health. These included the number of visits made to doctors' surgeries or hospitals in the previous six months, as well as all illnesses diagnosed, the number and type of treatments usually taken and the support or prosthetic devices used in daily life.

Based on the responses obtained, the questionnaire allowed the variables of personal finances, mental health and physical health to be scored on a scale of 1 to 6: excellent capacity, good capacity, slight deterioration, moderate deterioration, strong deterioration and total deterioration. For operational reasons, these categories were initially classified as good (excellent and good capacity), normal (slight and moderate deterioration) and poor (strong and total deterioration).

Given the scant frequency and lack of significance of the category "poor", however, only two categories, "good" and "normal-poor", were considered in the multivariate analysis.

### Measurement of independence

In the area of ADLs, the OARS questionnaire contains 7 items referring to basic or personal care activities (BADLs). These are eating and drinking, dressing/undressing, using toilets, walking unassisted (except with a stick), going to bed/getting up and bathing/showering. The questionnaire contains a further 7 items referring to instrumental activities (IADLs), namely using the telephone, mobility (travel), shopping, meal preparation, housework, medication management and money management.

After evaluating 14 items referring to ADLs, functional capacity was classified into 6 categories: excellent, good, slightly unsatisfactory, moderately unsatisfactory, seriously unsatisfactory and completely unsatisfactory. Our interest in this study was to study independent individuals against those presenting some degree of dependence, thus functional situation is classified into two categories. A score of one indicates good functional ADL ability, or independence to perform ADLs. A score of two is indicative of moderate-severe dependence in individuals who require assistance with some activities or need some help from another person every day to carry out ADLs.

### Statistical analysis

In the first place, the variables were described to permit a multivariate logistical regression analysis taking functional ADLs ability as the dependent variable. The choice of independent or predictive variables was made using a bivariate correlation matrix and Kendall's Tau-b correlation coefficient was used. The item exhibiting the highest correlation with the ADLs was considered the principal variable, while the remainder was treated as modifying variables. Where a high correlation was found between two independent variables, the measurement considered least reliable, most difficult to obtain or most subjective, was eliminated from the analysis [35]. In order to control collinearity, models were required not to have high correlation between predictor variables, and to have a Variable Inflation Factor (VIF) lower than 5 [36].

In the second place, we used a logistic regression model with the Backward Stepwise Wald Method. The goodness-of-fit of these models was studied by means of the Hosmer-Lemeshow test, which required > 0.05 in all cases. Among the estimators obtained from this analysis, Nagelkerke's R^2 ^coefficient, the Odds ratio with a confidence interval of 95%, and sensitivity and 1-specificity were employed to present the results in graphic form. Positive and negative predictive values were calculated to quantify the goodness of the model.

Missing information was not considered. The statistical analysis was performed using the SPSS 14.0 application. The study was evaluated and approved by the Ethics Committee at the Regional Government of Aragon, Spain (11/2009 minutes, 17^th ^June, 2009).

## Results

Of the population interviewed, 43.2% were men and 56.8% women. By age groups, 49.1% of respondents were between 65 and 74 years old and, 42.5% were between 74 and 84. Only 8.4% of the individuals frequenting social centres who responded to the questionnaire were aged 85 or older. The socio-demographic characteristics of the sample and the remaining variables are shown in Table 1.

**Table 1:** Socio-demographic characteristics, health and social resources

**Characteristics**		**Number**	**%**
**Gender**	Male	162	43.2

	Female	213	56.8

**Age**	Under 75	186	49.1

	75 and older	193	50.9

	Under 80	286	75.5

	80 and older	93	24.5

**Education**	Incomplete primary schooling	225	61.8

	Primary or higher schooling	139	38.2

**Marital status**	Unmarried/separated/divorced	45	12.8

	Married/stable partner	152	43.2

	Widowed	155	44

**Perceived health**	Good	224	63.6

	Normal or poor	128	36.4

**Personal finances**	Good	216	58.5

	Normal	148	40.1

	Poor	5	1.4

**Physical health**	Good	216	57.6

	Normal	145	38.7

	Poor	14	3.7

**Mental health**	Good	280	74.7

	Normal	76	20.3

	Poor	19	5.1

**ADLs**	Independent	239	63.7

	Moderate/Severely dependent	136	36.3

As reflected in Table 2, given that physical health exhibits the highest correlation with ADLs, this variable was considered the principal variable in the logistic regression analysis, while mental health, age, sex, marital status, education and personal finances were treated as modifying variables. This analysis was performed for two age groups, the first being under 75 and 75 years and older, and the second under 80 and 80 years and older. We shall call the first Model A, and the second Model B. Perceived health was not included in the model given its close linkage to the physical health variable, and in order to avoid collinearity problems.

**Table 2:** Bivariate correlation matrix for the variables considered in the study

	ADL	SEX	MARITAL STATUS	EDUCATION	PERCEIVED HEALTH	PERSONAL FINANCES	MENTAL HEALTH	PHYSICAL HEALTH	AGE (75 YEARS)	AGE (80 YEARS)
ADL	1.000 .001 375	.165(**) .002 347	.161(**) .000 374	-.139(**) .000 347	.367(**) .000 375	.100 .000 375	.530(**) .000 375	.576(**) .000 375	.287(**) .000 374	.236(**) .000 374

SEX		1.000 . 380	.114(*) .027 352	-.115(*) .028 364	.105(*) .050 352	.102 .051 369	.137(**) .008 375	.095 .067 375	.005 .915 379	.065 .207 379

MARITAL STATUS			1.000 . 352	-.121(*) .018 352	.083 .106 351	.034 .516 346	.103(*) .046 347	.077 .136 347	.243(**) .000 351	.245(**) .000 351

EDUCATION				1.000 . 364	-.192(**) .000 352	-.084 .112 358	-.178(**) .001 359	-.168(**) .001 359	-.138(**) .009 363	-.110 .037 363

PERCEIVED HEALTH					1.000 . 352	.238(**) .000 346	.186(**) .001 347	.470(**) .000 347	.042 .427 351	.007 .893 351

PERSONAL FINANCES						1.000 . 369	.150(**) .004 369	.129(*) .013 369	.011 .833 368	-.012 .816 368

MENTAL HEALTH							1.000 . 375	.456(**) .000 375	.240(**) .000 374	.275(**) .000 374

PHYSICAL HEALTH								1.000 . 375	.251(**) .000 374	.247(**) .000 374

* p < 0.05 **p < 0.01

### Model A

In the first step, all the independent variables were taken into consideration, as well as first order interactions between physical health and the remaining variables (Nagelkerke's R^2 ^= 0.481). Initially, all interactions were eliminated as they were not significant (R^2 ^= 0.466). Meanwhile, the variables education, marital status and personal finances (R^2 ^= 0.529) were eliminated in a three-step sequential process. This left physical health, mental health, age and sex as the variables in the final model (Table 3).

**Table 3:** Odds ratio for the predictor variables for functional deterioration in ADLs

	**MODEL A**		**MODEL B**	
	OR (CI 95%)	Sig.	OR (CI 95%)	Sig.

Physical health^a^	8.82 (4.96–15.70)	0.0001	9.21 (5.20 – 16.31)	0.0001

Mental health^b^	6.38 (3.31–12.31)	0.0001	6.70 (3.48 – 12.89)	0.0001

Sex^c^	1.95 (1.09–3.51)	0.026	1.82 (1.02 – 3.23)	0.042

Age^d^	2.24 (1.26 – 4.00)	0.006	1.38 (0.72 – 2.64)	0.33

Constant	-3.01		-2.64	

R^2^	0.529		0.514	

VIF	1.39		1.36	

^a ^Reference category: Good physical health.

^b ^Reference category: Good mental health.

^c ^Reference category: Male.

^d ^Reference category: under 75 (Model A), under 80 (Model B).

### Model B

After following an identical process (with the exception of the age-cut off, in this case 80 years), the correlation coefficient initially obtained was similar to the first model (Nagelkerke's R^2 ^= 0.470). After eliminating all non-significant interactions, the coefficient was 0.457. Once again, a sequential process was applied to eliminate all non-significant variables from the model. This left physical health, mental health, sex and age as the variables in the final model (R^2 ^= 0.514).

### Predictors of functional deterioration for ADLs

As shown in Table 3, the possession of normal/poor physical health means the likelihood of some degree of dependence is at least 4.96 higher than in individuals whose physical health is good. In the case of mental health, the likelihood is at least 3.31 higher. The age variable appears in third place as an explanatory factor. Thus, as age increases from under to over 75 years old, the risk of ADLs dependence rises by at least 1.26. With regard to sex, women are at slightly more risk of dependence than men.

In Model B, the risk of becoming to some degree dependent increases at least 5.2 times when physical health deteriorates, while the figures for mental health and sex are similar to those in Model A. Finally, the age of the individuals forming part of the sample ceases to be significant in this model.

**Figure 1:**
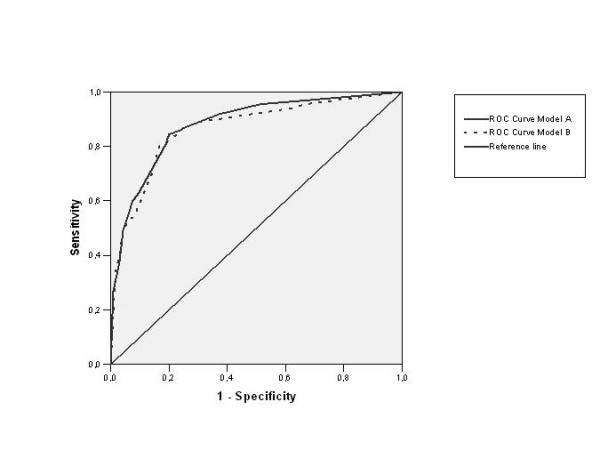
**ROC for ADL independence in models A and B**.

Figure 1 shows the ROC (Receiver Operating Characteristic) curves for the two models described. As can be seen, both exhibit high values (over 75%) in terms of sensitivity and specificity. Considering the sensitivity and specificity values for the different cut-off points, it may be observed that the optimum point in both models is 0.4. This cut-off point represents the highest value and it is located to the left on the ROC curve (Chart 1). Note that both curves are very similar for Models A and B. In fact, the area below the Model A curve is 0.880 (95% CI = 0.843 – 0.916) and that of Model B is 0.866 (95% CI = 0.826 – 0.906).

In addition, the negative predictive value for Model A is 0.88 whilst for Model B it is 0.92 (see Table 4). Thus, where the model indicates that the subject is independent, the prediction will only be erroneous 12% of the time in model A and 8% in model B. Thus, the second model represents an improvement on the first.

**Table 4:** Predictive values for the model used to determine functional dependence in ADLs.

	Sensitivity	Specificity	1-Sensit.	1-Specif.	Positive predictive value	Negative predictive value
Model A	0.822	0.808	0.178	0.192	0.724	0.88

Model B	0.800	0.833	0.200	0.176	0.68	0.92

Model A: Age two age group: under 75 and 75 years and older.

Model B: Age two age group: under 80 and 80 years and older

## Discussion

In this study we sought to obtain empirical evidence of successful ageing in individuals who maintain formal social relations on the assumption that the capacity to carry out the activities of daily living is a prerequisite for successful ageing. Furthermore, to the best of our knowledge, the target population selected is novel in the existing literature, insofar as all participants are still living at home and use the social resources already present in the community. In this context, 63.7% of the population exhibit complete independence in carrying out ADLs defined as basic and instrumental activities.

In view of the magnitude of this figure, it is important to identify the variables that determine, or help explain, independence among the population participating in formal social activity. As expected, the results of the logistic regression analysis performed suggest deterioration of physical and mental health, gender and age as predictors of disability or disability risk factors.

Physical health is the main predictor of disability in both models. In fact, deteriorating physical health was found to increase the risk of ADLs disability by at least 4.9 times. Guralnik and Simonsick showed that the presence of a single chronic condition is a significant predictor of deterioration in functional capacity, since such conditions are, in general, the main cause of difficulty in performing ADLs [37]. In Ho et al. five chronic conditions were analysed, allowing the researchers to conclude that individuals who have suffered a heart attack are at high risk of becoming dependent, if they are not already so [38].

Deteriorating mental health increases the risk of dependence by at least 3.3 times in our study. Similar results have been obtained by other authors [39-41], who found a strong association between levels of anxiety and functional capacity. Meanwhile, a significant association between the symptoms of depression and functional capacity has been noted [42].

Age and female gender are among the potential risk factor predictors for disability or associated with ADLs dependence [38,43-45]. Age is probably the key factor [46], increasing the risk of functional deterioration by 2.0 every ten years [37]. In our study, age is, indeed, a determining factor for disability, but above the age of 80 it loses significance as a predictor of deterioration, as may be observed in Model B. This is probably because the majority of the population of this age suffers from some level of dependence.

In terms of the sex variable, it is women who have the highest risk of developing disability problems, since gender appears as a risk factor in both models with significant OR values. Furthermore, these results are found regardless of age, given that there are no statistically significant differences between the mean age of men (74.4; CI = 73.4–75.4) and women (75.6; CI = 74.7–76.5). These results are close to the findings of other authors [1,38], who shows that ADLs independence at any given age was lower among women than among men, which reflects the superior longevity of women and the higher mortality of men at all ages. An explanation to these results could be that as long as men are fully independent, they live longer than women, but once their health begins to deteriorate, the progression of disability and the onset of death are faster in men [2]. This is because men suffer more fatal diseases like cardiovascular disease and strokes, whilst women tend to suffer from non-fatal but chronic disabling conditions like arthritis and osteoporosis. However, other scholars have argued that this difference disappears if other factors such as chronic diseases are also controlled [46], and it has been suggested the incidence of dependence is in fact similar taking into consideration the longer life expectancy of the female population [47].

The marital status, education and personal finances variables did not turn out to be significant disability risk predictors. Nevertheless, other authors have found that married people tend to function better than single or widowed individuals [48]; that there is a direct relationship between the number of years people can live independently and their level of education [1,37,49]; and that the likelihood of functional deterioration increases almost three times among individuals with low incomes [49].

One of our most interesting findings is that the model employed has considerable predictive power with regard to independence. Thus, it is relatively unlikely that an independent individual will not be identified as such by the model. In fact, only between 12% and 8% of individuals (depending on the age range considered) would be in this situation. In this light, the model may be interpreted as explaining a significant higher part of ADL disability than the models employed in other, similar studies. However, the advantages of the questionnaire and the model employed need to be considered in light of the difficulties inherent in the data gathering procedure.

The proportion of the population with independence in performing ADLs in our work (63.7%) is higher than the percentages obtained in studies performed in other Spanish cities using identical methodology. Martínez de la Iglesia et al. obtained a figure of 49% [50]; Eiroa et al. found that 57.6% of the population was sufficiently independent to carry out ADLs [51]; and Azpiazu et al. found that this percentage was 58% [52]. All four studies used the multidimensional OARS to measure the functional capacity of non-institutionalised old people, but only our study features social participation by all of the respondents in the survey.

Comparison with results of these national studies allows the conclusion to be reached that, in terms of the Verbrugge and Jette disability model, participation by the elderly population in social centre activities is an external or environmental factor that acts as a delaying mechanism for functional dependence [53].

This statement seems to agree with results from the literature concerned with the relation between social networks of the elderly population and the functional capacity of this group to perform ADLs.

Two studies have recently been published in Spain regarding the relation between social networks of the elderly population, whether informal or formal relationships, and ADL disability [27,54]. By means of an index of social engagement in community and family activities in the older population of the city of Leganés, the first study concludes that social engagement is associated with a lower prevalence of ADL disability. [27]. The second analyses the effect of social networks on the level of autonomy (instrumental and basic disability) at the initial stages of old age, in two metropolitan areas. By means of two indicators, a general one for social network and another for involvement, it is found that social engagement has strong influence in delaying the onset of disability, for both BADLs and IADLs, although to a greater degree for basic activities [54].

Among international studies, Mendes de Leon et al. in North America concluded that leisure activities and contact with other persons reduce the risk of disability [55]. Similarly, in the Avlund et al. study for the Nordic countries, diversity in social contacts and high social participation predicted the maintenance of basic activities of daily living [56]. More recently, evidence that diversity of social ties are beneficial with respect to prevalence and recovery from ADL disability among older people in three European countries (CLESA Project), and with respect to four basic activities of daily living [27].

A different analysis is that used for the socially-active elderly (with participation in groups of elderly people or engaged in activities outside the nuclear family) in the metropolitan region of Porto Alegre [14]. Among independent predictive factors of successful ageing are independence in performing daily life activities and autonomy. Consequently, it seems that the adoption of independence in ADLs as a proxy for successful ageing would not be an absurd notion.

The described works appear to agree that social relations of elderly people, considered on the whole, are beneficial for the maintenance of independence and functional capacity for performing ADLs. However, not all factors covered by social relations contribute in the same degree [26,54]; rather, formal social engagement, more than the family network, has a greater protective effect.

Another reading of the results of this work shows that there is a significant proportion (36.3%) of elderly people who regularly frequent these social centres despite presenting a degree of ADL dependency. Dependency, at least in its least severe manifestations, is no obstacle for maintaining formal social relations and participating in both recreational and educational activities.

Although our results are in line with the described published studies, there are two fundamental weaknesses: the first is the adoption of a transversal design that does not allow a relation of causality be established between social engagement and dependence for ADLs, and the second is that this work was only made with socially-active older people.

However, in this regard, the study presented here may be considered as a starting point. To take this research further, it would interesting to apply this method to non-institutionalised subjects who do not take part in social centres in order to examine differences in the results obtained for both populations. Also, a longitudinal analysis of the population aged 65 and over, with and without participation in social centres, would allow clarification of the risk factors explaining the loss of independence in both populations. This would provide a better understanding of the interrelationship between social engagement and successful ageing.

## Conclusion

Given the high percentage of independent elderly persons in this study, the high predictive power of the model with regard to independence, the fact that community participation in social activities seems to be beneficial as a protective mechanism against functional dependence, and that deterioration in physical and mental health is the main controllable cause of functional disability, public policies should foster this type of centre and develop health promotion programmes.

## Competing interests

The authors declare that they have no competing interests.

## Authors' contributions

ER conceived the study, performed the statistical analysis, interpreted findings and drafted the manuscript. AL conceived the study, interpreted findings and drafted the manuscript. ASS interpreted findings and drafted the manuscript.

## Pre-publication history

The pre-publication history for this paper can be accessed here:


